# Postvaccination COVID-19 among Healthcare Workers, Israel

**DOI:** 10.3201/eid2704.210016

**Published:** 2021-04

**Authors:** Sharon Amit, Sharon Alexsandra Beni, Asaf Biber, Amir Grinberg, Eyal Leshem, Gili Regev-Yochay

**Affiliations:** Authors affiliation: Chaim Sheba Medical Center, Ramat-Gan, Israel

**Keywords:** vaccination, healthcare workers, respiratory infections, severe acute respiratory syndrome coronavirus 2, SARS-CoV-2, SARS, COVID-19, coronavirus disease, zoonoses, viruses, coronavirus, Israel, vaccines

## Abstract

Coronavirus disease (COVID-19) symptoms can be mistaken for vaccine-related side effects during initial days after immunization. Among 4,081 vaccinated healthcare workers in Israel, 22 (0.54%) developed COVID-19 from 1–10 days (median 3.5 days) after immunization. Clinicians should not dismiss postvaccination symptoms as vaccine-related and should promptly test for COVID-19.

Large-scale vaccination of risk groups and later the general population is the single most effective public health measure for mitigation of the coronavirus disease (COVID-19) pandemic. National COVID-19 vaccination programs started during December 2020 in several countries and prioritized healthcare workers (HCWs) (*1*). In some countries the vaccination programs coincided with a surge in detected COVID-19 cases and increased burden on the healthcare system (*2*). 

During December 2020–January 2021, Israel experienced a surge in COVID-19 incidence that resulted in the third national lockdown imposed since the pandemic began in early 2020 (*3*). Concomitantly, during December 2020, Israel’s Ministry of Health approved the Pfizer-BioNTech COVID-19 vaccine (BNT162b2; Pfizer Inc., https://www.pfizer.com) and prioritized HCWs for immunization (*4*). 

Sheba Medical Center is a large hospital with 9,069 staff members in Ramat-Gan, Israel. The hospital started its personnel vaccination program on December 20, 2020, and excluded workers who had recovered from COVID-19. During the first week of the campaign, 4,081 (45%) eligible staff members received the first dose of BNT162b2. Concurrently, the national COVID-19 positivity rate rapidly increased to >6% on January 3, 2021 (*2*). 

## The Study

The hospital’s Infection Prevention and Control Unit conducted active and passive surveillance of vaccinated staff by using daily health questionnaires, hotlines, on-call infectious disease unit staff, and post-vaccination web-based questionnaires to identify and test symptomatic HCWs. Among 4,081 HCWs vaccinated in the first week of the campaign, 22 (0.54%) later had laboratory-confirmed COVID-19 (Table). The average age among COVID-19–positive vaccinated HCWs was 45.3 years (±9.85 years), and they belonged to different healthcare sectors and worked on various wards. 

**Table:** Coronavirus disease cases among healthcare workers in the early postvaccination period, Israel, December 20, 2020–January 2, 2021*

Case no.	Age, y/sex	Ward	Healthcare sector	Indication for testing	Presumed exposure source	Exposure day	Day of symptom onset†	Day tested	No. days from symptom onset to testing	No. secondary isolations
1	42/F	General surgery	Physician	Symptoms	Unknown	Unknown	−4	+5	Excluded‡	0
2	54/F	Transportation	Secretary	Symptoms	Unknown	Unknown	0	+9	9	1
3	34/M	Geriatrics	Physician	Symptoms	Unknown	Unknown	+1	+1	0	1
4	31/F	Cardiovascular surgery	Nurse	Symptoms	Community	Unknown	+1	+1	0	1
5	49/F	Psychiatry	Cleaning services	Symptoms	Unknown	Unknown	+1	+3	2	0
6	43/F	Laundry	Laundry handler	Symptoms	Community	−3	+2	+3	1	3
7	43/F	Laboratory	Scientist	Exposure	Community	−3	+2	+6	4	0
8	60/F	ED	Nurse assistant	Symptoms	Community	0	+3	+5	2	0
9	50/F	Eye clinic	Technologist	Symptoms	Unknown	Unknown	+4	+6	2	0
10	33/M	Psychiatry	Psychologist	Exposure	Community	+2	+6	+6	0	0
11	36/M	Operating room	Logistics	Symptoms	Community	+4	+7	+8	1	0
12	54/M	Pulmonology	Physician	Symptoms	Community	+4	+7	+7	0	0
13	37/M	ED	Physician	Symptoms	Unknown	+3	+7	+9	2	0
14	32/M	Rehabilitation	Nurse	Symptoms	Unknown	Unknown	+9	+10	1	1
15	40/M	Laboratory	Physician	Symptoms	Unknown	Unknown	+10	+10	0	1
16	52/F	Radiotherapy	Secretary	Exposure	Unknown	Unknown	Asymp	+5	NA	3
17	55/F	General surgery	Phlebotomist	Exposure	Unknown	Unknown	Asymp	+8	NA	4
18	55/F	Kitchen	Food handler	Exposure	Community	−5	Asymp	+2	NA	1
19	61/F	Radiology	Physician	Exposure	Community	+4	Asymp	+11	NA	0
20	40/F	ED	Secretary	Exposure	Community	+6	Asymp	+11	NA	2
21	45/F	Internal medicine	Nurse	Exposure	Unknown	Unknown	Asymp	+8	NA	0
22	39/M	Internal medicine	Nurse	Exposure	Community	+2	Asymp	+8	NA	0

*All persons with cases were vaccinated during the first week of campaign, December 20–27, 2020. Asymp, asymptomatic; ED, emergency department; NA, not applicable. †Considering day of vaccination as day 0. ‡Excluded from calculations of mean time from vaccination to symptom onset because symptoms began before vaccination.

Among the 22 vaccinated HCWs who tested positive for COVID-19, 13 were tested because they had symptoms, most commonly an influenza-like illness that included fever, chills, cough, headache, myalgia, and sore throat. Two vaccinated HCWs were tested because of exposure to confirmed or suspected COVID-19 cases and because they reported symptoms upon questioning. Asymptomatic COVID-19 cases were identified among HCWs as part of postexposure screening. Among the 22 COVID-19–positive HCWs, 11 had presumable community-related exposures, 4 of whom reported exposure incidents that occurred before or on the date of vaccination. An investigation conducted by the hospital’s Infection Control and Prevention Unit identified 10 healthcare-related secondary exposures. However, we did not identify any point-source exposures or COVID-19 clusters linked to the immunization process.

Among the 11 vaccinated HCWs who reported COVID-19 symptoms, the median time between the first dose of BNT162b2 immunization and symptom onset was 3.5 (range 0–10) days; we excluded 1 vaccinee from our calculation and analysis because the HCW had symptoms before immunization (Table). The median time between the onset of symptoms and testing was 1 day, demonstrating the high level of suspicion for COVID-19 during the vaccination campaign. 

Of note, apart from the need for early detection, persons who test positive for COVID-19 after receiving the first vaccine dose (whether asymptomatic and tested following exposure or tested because they are symptomatic) are not eligible to receive the second dose, according to Ministry of Health policy. However, depending on availability of vaccines, this policy might change when further data are collected.

## Conclusions

COVID-19 in HCWs is a major concern for health authorities worldwide. HCWs, especially acute and chronic care facility personnel, are at high risk for contracting symptomatic and asymptomatic COVID-19 and might become infected at home or nosocomially while caring for patients or interacting with other staff members (*5*–*7*). Infections among HCWs have an immediate effect on their close occupational environment and the overall healthcare system. Secondary exposures, isolation, and infections of staff can substantially impair the capacity of a single ward to care for patients, creating a snowball effect with collateral damage to both the functional resilience of the facility and morale of staff. Consequently, as soon as COVID-19 vaccines were deployed in Israel, HCWs were the first group to receive it. 

We report 22 cases of early, postimmunization, laboratory-confirmed COVID-19 among HCWs during the launch of the vaccination campaign in a large hospital in Israel. BNT162b2 is not likely to exert protection against clinical disease during the first days after receipt of the first dose. Efficacy of the BNT162b was 52% a week after the first dose, and positive COVID-19 cases were described among vaccinees even early after the second dose (*8*). Thus, during a large-scale immunization campaign coinciding with rapid national increase in COVID-19 cases, some immunized persons likely will develop clinical disease.

The co-occurrence of vaccination deployment with the rapidly climbing COVID-19 spread in many parts of the world is a confusing period in which hope is mixed with great vulnerability. The phenomenon of pandemic fatigue, in which the population tires of constant safety precautions, testing, isolation, and restrictions, could lead to less social distancing and personal protection. Pandemic fatigue coupled with the availability of a vaccine, might give the population a false sense of reassurance and consequently lead to a brisk increase in COVID-19 cases. Thus, almost every physical complaint after vaccination poses a true diagnostic dilemma as to whether an adverse reaction or a new COVID-19 infection is the cause. Undetected COVID-19 cases among HCWs could be hazardous for patients and other staff. 

Clinicians should have a high level of suspicion of reported symptoms and avoid dismissing complaints as vaccine-related until true infection is ruled out and vaccinees are tested. Active and passive surveillance schemes that enable rapid testing and initiation of infection control measures are essential in preventing possible diagnostic delays and secondary exposures. Therefore, healthcare-related indications for testing should not be altered until systematic and exhaustive data are gathered regarding vaccine effectiveness in healthcare settings.

## References

[1] Dooling K, McClung N, Chamberland M, Marin M, Wallace M, Bell BP, et al. The Advisory Committee on Immunization Practices’ interim recommendation for allocating initial supplies of COVID-19 vaccine—United States, 2020. MMWR Morb Mortal Wkly Rep. 2020;69:1857–9. 10.15585/mmwr.mm6949e1 33301429 PMC7737687

[2] Johns Hopkins University Center for Systems Science and Engineering. COVID-19 dashboard. 2020 [cited 2021 Jan 3]. https://coronavirus.jhu.edu/map.html 10.1016/S1473-3099(22)00434-0 PMC9432867 36057267

[3] Leshem E, Afek A, Kreiss Y. Buying time with COVID-19 outbreak response, Israel. Emerg Infect Dis. 2020;26:2251–3. 10.3201/eid2609.201476 32620179 PMC7454065

[4] State of Israel Ministry of Health. Coronavirus (COVID-19) vaccines [in Hebrew]. 2020 [cited 2021 Jan 3]. https://www.health.gov.il/UnitsOffice/HD/PH/epidemiology/td/docs/365_Corona.pdf

[5] Calcagno A, Ghisetti V, Emanuele T, Trunfio M, Faraoni S, Boglione L, et al. Risk for SARS-CoV-2 infection in healthcare workers, Turin, Italy. Emerg Infect Dis. 2021;27:303–5. 10.3201/eid2701.203027 33021927 PMC7774556

[6] Feaster M, Goh YY. High proportion of asymptomatic SARS-CoV-2 infections in 9 long-term care facilities, Pasadena, California, USA, April 2020. Emerg Infect Dis. 2020;26:2416–9. 10.3201/eid2610.202694 32614768 PMC7510707

[7] Akinbami LJ, Vuong N, Petersen LR, Sami S, Patel A, Lukacs SL, et al. SARS-CoV-2 seroprevalence among healthcare, first response, and public safety personnel, Detroit metropolitan area, Michigan, USA, May–June 2020. Emerg Infect Dis. 2020;26:2863–71. 10.3201/eid2612.203764 32956614 PMC7706918

[8] Polack FP, Thomas SJ, Kitchin N, Absalon J, Gurtman A, Lockhart S, et al.; C4591001 Clinical Trial Group. Safety and Efficacy of the BNT162b2 mRNA Covid-19 Vaccine. N Engl J Med. 2020;383:2603–15. 10.1056/NEJMoa2034577 33301246 PMC7745181

